# Sex and gender differences in access, quality of care, and effectiveness of treatment in dementia: a scoping review of studies up to 2024

**DOI:** 10.1186/s13690-025-01626-z

**Published:** 2025-05-29

**Authors:** Elisa Aguzzoli, Magdalena Walbaum, Martin Knapp, Laura Castro-Aldrete, Antonella Santuccione Chadha, Eva Cyhlarova

**Affiliations:** 1 Care Policy and Evaluation Centre, London, School of Economics and Political Science, London, WC2A 2AE UK; 2 Women’s Brain Foundation, 4052 Basel, Switzerland

**Keywords:** Dementia, Sex and gender differences, Quality of care, Treatments, DMTs

## Abstract

**Background:**

Dementia represents one of the greatest global health challenges. Women have a greater lifetime risk of developing dementia compared to men. Both pharmacological and non-pharmacological interventions aimed at slowing cognitive decline show promising results. However, most studies do not examine whether there are sex and gender differences in access to treatment, quality of care or treatment effectiveness.

**Objectives:**

To summarise evidence on sex and gender differences in access to treatment, management, and treatment effectiveness for people with dementia.

**Methods:**

This scoping review followed PRISMA guidelines and was conducted in PubMed/MEDLINE, EMBASE, Google Scholar, and ClinicalTrials.Gov databases in November 2023 and updated in January 2024. Systematic reviews and observational studies were included to explore sex and gender differences in access or management of dementia. Systematic reviews and clinical trials were included to investigate sex and gender differences in treatment effectiveness.

**Results:**

We included 16 studies in our review. Sex and gender differences were observed in the prescription and receipt of anti-dementia medications, as well as access to primary care, with variations by settings. Mixed results were found concerning polypharmacy and inappropriate medications, with women being prescribed antipsychotic and other psychotropic medications for longer periods compared to men. Studies of the impact of exercise on cognitive decline yielded mixed results, though limited disaggregated data by sex indicated a more pronounced impact in women than in men. Cognitive stimulation therapy interventions showed greater cognitive improvements for women. Clinical trials assessing the effectiveness of disease-modifying therapies (DMTs) suggest that women may be less responsive to DMTs than men.

**Conclusions:**

There are important differences between men and women living with dementia in access to diagnosis, treatments, quality of care and effectiveness of treatments. Such differences can significantly impact health outcomes. Sex and gender inequalities should be considered when planning and implementing healthcare, social care, and associated strategies in dementia. To provide conclusive evidence, it is essential for clinical trials to have sufficient statistical power and report outcomes disaggregated by sex.

**Supplementary Information:**

The online version contains supplementary material available at 10.1186/s13690-025-01626-z.

## Highlights

• Women have a greater lifetime risk of developing dementia than men.

• Sex and gender differences in access to treatment, quality of care or treatment effectiveness in dementia have been overlooked in research.

• Sex and gender differences were observed in the prescription and receipt of anti-dementia medications, as well as access to primary care, with variations by settings.

• Women with dementia are prescribed antipsychotic and other psychotropic medications for longer periods compared to men.

• Cognitive stimulation therapy shows greater cognitive improvements for women.

• Clinical trials assessing the effectiveness of disease-modifying therapies (DMTs) suggest that women may be less responsive to DMTs than men.

• Sex and gender inequalities should be considered when planning and implementing dementia strategies and care policies.

**Supplementary Information:**

The online version contains supplementary material available at 10.1186/s13690-025-01626-z.

## Abstract (plain language summary)

Dementia represents one of the greatest global health challenges. Women have a greater lifetime risk of developing dementia compared to men. There is substantial evidence that pharmacological and non-pharmacological interventions can slow cognitive decline or improve quality of life of people with dementia. However, most studies overlook sex and gender differences in access to treatment, quality of care, and effectiveness. The aim of this scoping review is to summarise evidence on sex and gender differences in access to treatment and care, management, and treatment effectiveness. We followed standard and published methods to conduct our review. We found that there are sex and gender differences in the prescription and receipt of anti-dementia medications, as well as access to primary care. Mixed results were found concerning the use of medications inappropriately, with women being prescribed antipsychotic and other psychotropic medications for longer periods and with less monitoring compared to men. Studies of the impact of exercise on cognitive decline showed mixed results, though limited disaggregated data by sex indicated a greater impact in women compared to men. Cognitive stimulation therapy showed greater cognitive improvements for women. Additionally, clinical trials assessing the effectiveness of disease-modifying therapies suggest that women may be less responsive to these new medications compared to men. In conclusion, there are important differences between men and women with dementia in access to diagnosis, quality of care and treatment effectiveness. Sex and gender inequalities should be considered when planning and implementing healthcare, social care, and associated strategies for dementia.

**Supplementary Information:**

The online version contains supplementary material available at 10.1186/s13690-025-01626-z.

**Table:** 

Text box 1. Contributions to the literature
• This study highlights important differences between men and women living with dementia in access to diagnosis, treatments, quality of care and effectiveness of treatments. Such differences can significantly impact health outcomes.
• It also reveals an existing gap in the evidence regarding differential effectiveness of interventions by sex and gender, thus limiting effective planning and strategies related to dementia care.
• These findings emphasise the critical need to report disaggregated outcomes by sex and gender in dementia research.

## Background

Brain health is key to everyone’s life since it shapes individual development, quality of life, and overall wellness. Among the main brain health disorders, dementia represents one of the greatest global and public health challenges [1]. It is estimated that, in 2019, there were over 55 million people with dementia globally; this number is predicted to increase rapidly over the coming decades [2]. In 2019, the prevalence of dementia among older people in the UK was estimated at 7.1% [3], that is around 900,000 people living with dementia in the UK [3]. This number is expected to rise to 1.6 million by 2040 [3]. Depending on the type of dementia, individuals may experience various impacts on cognition, mental health, agitation, communication, mobility, and behaviour. Dementia can also have significant impacts on family members and other unpaid carers, often women [4, 5], and on society as a whole.

Clinical trials of interventions aiming to prevent and/or delay dementia onset and slow its progression have been conducted over recent decades with mixed results. Pharmaceutical companies have re-directed considerable resources to research and development, aspiring to find effective treatments to prevent or reduce the progression of cognitive decline and other symptoms among the older population. Three acetylcholinesterase inhibitors (AChEIs; donepezil, galantamine, and rivastigmine) and memantine have been shown to slow cognitive deterioration in people with Alzheimer’s disease (AD) and are considered ‘usual care’ for this group of patients in the UK [6–8]. These pharmacological treatments, prescribed by dementia specialists after diagnosis, are the only available anti-dementia medications currently approved for use in the UK [8]. Antipsychotic medications are sometimes used to manage symptoms like agitation and psychosis, but their use is not considered appropriate because they increase the risk of falls, diabetes, heart disease and mortality in a dementia population [9].

There are currently 164 trials of AD treatments at different phases worldwide, assessing 127 unique treatments. Disease-modifying treatments (DMTs) are the most extensively studied today, comprising 75% of the drugs under investigation in clinical trials, including disease-modifying biological agents and disease-modifying small molecule drugs [10]. To date, aducanumab and lecanemab have been approved by the Food and Drug Administration (FDA) in the USA [11–16], while only lecanemab has, at the time of writing, been approved—by the European Medicines Agency (EMA) [17]. None has been approved yet by the Medicines and Healthcare products Regulatory Agency (MHRA) in the UK. These agents are currently under review by other regulatory bodies in Japan and China [18, 19].

Several factors contribute to sex and gender differences in dementia. Sex refers to the biological characteristics, such as physical and physiological features, that distinguish females and males. On the other hand, gender is a socio-cultural construct that encompasses behaviours, roles, and self-identification shaped by societal norms and cultural perceptions of what it means to be a woman or a man [20–22]. In this review, we use the terms women/men and female/male interchangeably to align with the terminology used in the existing literature and to reflect the overlap in how these concepts are discussed in research. Globally, in 2019, it was estimated that the female-to-male ratio of people with dementia was 1.69 [2]. Age is one of the major dementia risk factors [23, 24] and, as women generally live longer, they are at greater lifetime risk of developing dementia [22]. For example, women face nearly twice the risk of developing AD compared to men [21]. Some studies suggest that hormonal changes and menopause could increase the risk of developing AD [25]. Other reasons for differences between men and women are linked to known risk factors, such as inequalities in early life education, which disproportionally affect women [26], or higher rates of smoking, coronary artery disease, and brain injury with loss of consciousness in men [21]. Additionally, inequalities in diagnosis and treatment contribute to differences in AD progression [27]. Women are often diagnosed at later stages, leading to delayed management and more rapid decline after diagnosis than men [28]. Furthermore, disparities exist in the ongoing monitoring and management of the condition [29].

Potential sex and gender differences in AD treatment effectiveness have been attributed to differences in drug pharmacokinetics, pharmacodynamics, and metabolism between men and women [30]. Some studies suggest that there are no significant differences in pharmacological benefits [6] or treatment effectiveness between men and women [7, 31–33]. However, others argue that the absence of data disaggregated by sex and gender on treatment effectiveness makes it difficult to draw definitive conclusions [34–36]. The lack of clear evidence on sex and gender differences in treatment effectiveness is partly due to the under-representation of women in clinical trials for AD treatment. Studies have shown that the proportion of women in randomised controlled trials (RCTs) of experimental drugs does not reflect their proportion in the dementia population [37].

## Objectives

The aim of this scoping review is to summarise evidence on sex and gender differences in AD and other dementias, focusing on differences in access, management, and treatment effectiveness. Given the potential impact of health and social care (sometimes called long-term care) service configurations and funding arrangements on patterns of access, management and effectiveness, we were particularly interested in mapping intervention studies and evidence for or relevant to the UK.

## Methods

### Search strategy

This scoping review followed the Preferred Reporting Items for Systematic Review and Meta-Analysis (PRISMA) guidelines. The literature search was run in PubMed/MEDLINE and EMBASE in November 2023 and updated in January 2024. We also conducted a targeted search of the grey literature using Google Scholar and ClinicalTrials.Gov. The search strategy design was guided by previous literature and by an extensive literature mapping conducted prior to this review. We used the same key terms for each database search to ensure consistency and relevance in the retrieved articles. Initially, only UK-based studies were selected; however, due to the limited number of studies, the search was expanded. Full details of the search strategies are presented in Supplementary Table 1.

### Eligibility criteria

Systematic reviews and observational studies were considered eligible for exploring sex and gender differences in access or management of dementia. Systematic reviews and clinical trials were eligible for investigating sex and gender differences in treatment effectiveness. In our study, we searched for studies including sex- or gender-disaggregated data. Previous studies have used both terminologies, hence, in this study we will refer to both. We excluded phase I and phase II trials because the treatments were either undergoing testing procedures and have not received approval by any regulatory agency, or they were discontinued before completing phase III. Eligibility was restricted to full-text papers published from 2010 onwards. Only articles published in English, Spanish or Italian were included.

### Study selection

Titles and abstracts of studies identified using the search strategy were screened by two reviewers. Full-text articles were assessed for potential eligibility by one reviewer (EA) and independently checked by a second reviewer (MW). Any disagreements were resolved by discussion or arbitration between the two reviewers.

### Data extraction

One independent reviewer extracted data from the selected studies by searching for sex and gender-disaggregated data in the results sections and the supplementary material. Study characteristics were summarised in Microsoft Excel spreadsheet as follows: author and date of publication, study design, sample size, study interventions, outcomes included, subgroup analyses, and study results.

### Data synthesis and critical appraisal

The evidence was categorised based on the type of interventions and/or dementia treatment analysed in each study. Study characteristics and findings on sex and gender differences were tabulated and categorised accordingly. We conducted critical appraisal of the included studies, resolving disagreements through discussion. We concisely summarised the data across multiple studies in a narrative synthesis while we ensured to convey and clarify meaning [38]. Narrative synthesis aims to develop a coherent narrative that summarises and describes evidence [39].

## Results

**Fig. 1:**
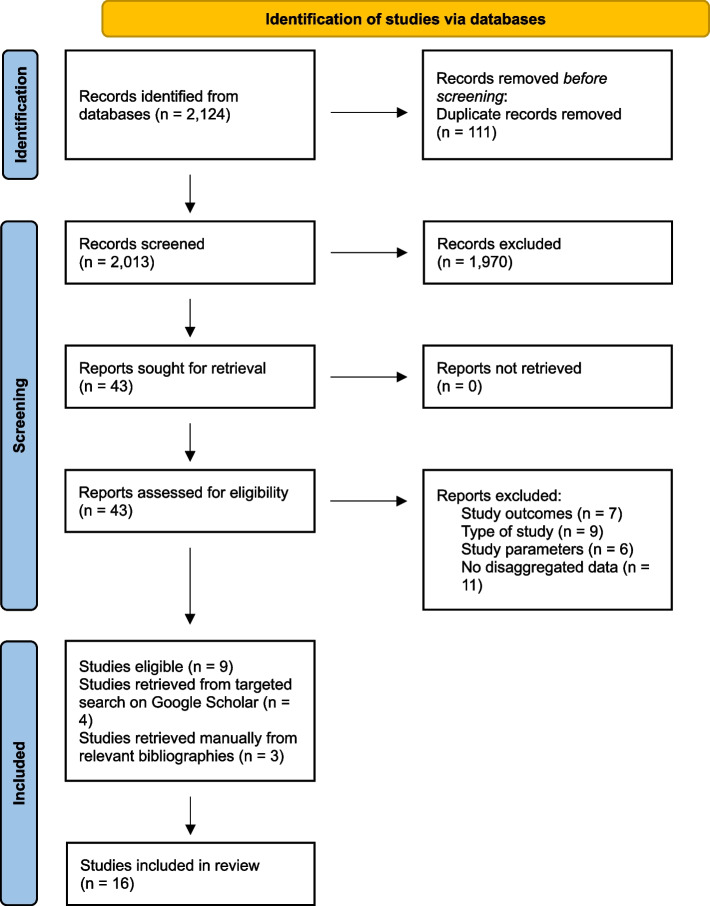
PRISMA flow diagram of study selection for the scoping review on sex and gender differences in access to treatment, quality of care, and medication prescription in dementia (literature up to 2024)

The initial search returned 2,124 articles. After removing duplicates, we identified 2,013 eligible articles (see Fig. 1). Screening of titles and abstracts of these records resulted in 43 studies being selected for full-text review. For the targeted search in the grey literature through Google Scholar, we reviewed the first 20 pages, with ten records per page. We also manually retrieved eligible studies from references listed in relevant studies. A total of 16 studies were included in the review (see Tables 1 and 2), nine studies were retrieved from PubMed/MEDLINE, EMBASE and ClinicalTrials.Gov, four from Google Scholar and three manually retrieved. Reasons for exclusion at the full-text stage are presented in Fig. 1.

**Table 1:** Characteristics of studies included in the scoping review on sex and gender differences in access to treatment, quality of care, and medication prescription in dementia (literature published up to January 2024)

Author	Year	Country	Design of the study	Sample size	Intervention	Main outcome	Results
Cooper et al	2015	UK	Analysis of primary care records from The Health Improvement Network (THIN) database (2002–2013)	77,045 people with dementia	Access to anti-dementia medication	Inequalities in anti-dementia medication prescribing	Women with dementia were less likely to be initiated on anti-dementia drugs compared to men (IRR 0.96; 95% CI 0.94–0.98)
Lu et al	2021	US	Retrospective cross-sectional study	1,240 Medicare beneficiaries	Use of anti-dementia medication	Gender disparity in anti-dementia medications	Women were more likely to receive anti-dementia medications for AD and related dementias (OR 1.71; 95% CI 1.19–2.45) and AD-related dementias (OR 1.90; 95% CI 1.23–2.95), but not statistically significant difference for AD only (OR 1.20; 95% CI 0.58–2.47)
Cooper et al	2016	UK	Analysis of primary care records from THIN database (2002–2013)	68,061 people with dementia	Use of mental and physical healthcare	Prescribing attitudes and sex and gender differences	Women had lower rates of surgery consultations (IRR 0.90, 95% CI 0.90–0.91), annual blood pressure monitoring (IRR 0.96, 95% CI 0.95–0.97) and annual weight monitoring (IRR 0.91, 95% CI 0.90–0.93) Women were more likely to be prescribed antipsychotics (IRR 1.18; 95% CI 1.13–1.23), hypnotics (IRR 1.06; 95% CI 1.02–1.12) and anxiolytics (IRR 1.13; 95% CI 1.07–1.19)
Jones et al	2020	UK	Analysis of primary care records from THIN database (2014–2016)	53,718 people with dementia	Antipsychotic drugs prescription	Psychotropic drug prescribing initiation and duration	Women were less likely to be prescribed antipsychotics (PRR 0.95; 95% CI 0.92–0.99), but there was a non-significant difference with anxiolytics/hypnotics (PRR 0.99; 95% CI 0.95–1.02)
Montastruc et al	2013	France	Analysis of French cohort REAL.FR (2000–2002)	684 people with AD	Potentially inappropriate medication (PIM) use	PIM use	Women were more likely to be using at least one PIM (OR 1.5; 95% CI 1.1–2.2)
Stephens et al	2014	UK	Analysis of IMS Health’s Hospital Treatment Insights database (2010–2012)	63,079 inpatients with dementia	Antipsychotic drugs prescription	Antipsychotic drug prescribing during in hospital care	Men were more likely to be prescribed antipsychotics during hospital stay (OR 1.1; 95% CI 1.06–1.15),
Stocks et al	2017	UK	Analysis of the CPRD primary care database (2001–2014)	111,346 patients	Antipsychotic drugs prescription	Temporal changes in the prescribing of antipsychotic drugs	Women with dementia without a psychotic disorder diagnosis were more likely to receive repeated antipsychotic prescriptions, although not statistically significant (OR 1.06; 95% CI 0.95–1.17)
Sourial et al	2020	Canada	Secondary analysis of a retrospective chart review	N/A	Implementation of the Quebec Alzheimer Plan (QAP)	Quality of care as measured by a score based on consensus guidelines	Improvements in the quality of dementia care were larger for men than women (mean difference 4.97; 95% CI 0.08, 9.85)
Tjia et al	2010	Germany	Secondary analysis of CASCADE Study	572 nursing home residents with advanced dementia	Use of inappropriate medication	Use of medications classified as never appropriate in advanced dementia	Men with advanced dementia living in nursing homes were significantly more likely to be prescribed with medications classified as never appropriate for use in advanced dementia (OR 2.52; 95% CI 1.21–5.27)

*OR* Odds ratio, *CI* Confidence Interval, *IRR* Incidence Rate Ratio, *PRR* Prevalence Rate Ratio, *PIM* Potentially Inappropriate Medication, *CPRD* Clinical Practice Research Datalink

**Table 2:** Characteristics of studies included in the scoping review on sex and gender differences in treatment effectiveness for people with dementia (literature published up to January 2024)

Author	Year	Country	Design of the study	Sample size	Intervention	Primary Outcome	Summary of results
Baker et al	2010	US	Randomised controlled trial	33 participants (17 females)	2:1 ratio to aerobic exercise or stretching control groups. Duration: 6 months	Symbol-Digit Modalities, Verbal Fluency, Stroop, Trails B, Task Switching, Story Recall, and List Learning	Favourable effects of aerobic exercise were apparent for Symbol-Digit Modalities (*p* = 0.05) and Verbal Fluency (*p* = 0.04). The effect size magnitude was larger for women than men on both tasks (symbol-digit: f_women_ = 0.67, *p* = 0.04; f_men_ = 0.29, *p* = 0.33; category fluency: f_women_ = 0.88, *p* = 0.01; f_men_ = 0.28, *p* = 0.39)
Lamb et al	2018	UK	Multicentre, pragmatic, investigator masked, randomised controlled trial	494 participants (329 exercise programme and 165 usual care)	4 months of supervised exercise and support for ongoing physical activity, or usual care only Duration: 12 months	Cognitive decline (measured with ADAS-Cog 12)	High intensity exercise was not effective in reducing cognitive decline (adjusted mean difference -1.4; 95% CI -2.6 to -0.2). Non-significant bigger difference in women than men when comparing treatment versus usual care (adjusted mean difference -1.8; 95% CI -3.60 to 0.08 and -1.2; 95% CI -2.78 to 0.46, respectively)
Lawlor et al	2018	9 European countries	Phase III randomised controlled clinical trial	511 participants (258 placebo, 253 nilvadipine)	Sustained-release nilvadipine Duration: 78 weeks	Cognitive decline (measured with ADAS-Cog 12)	No treatment benefit for nilvadipine on ADAS-Cog 12 (*p* = 0.465). No statistically significant difference between men and women: ADAS-Cog12 scores adjusted mean difference -1.95; 95% CI (-4.91 to 1.0) in men versus 0.84; 95% CI (-1.42 to 3.11) in women
Aguirre et al	2013	UK	Randomised controlled trial	272 participants	14 sessions of 45-min of Cognitive stimulation Duration: 7 weeks	Cognitive decline (measured with ADAS-Cog)	CST has cognitive benefit for people with dementia. Greater improvements in cognition were associated with female gender (ADAS-Cog score F = 5.1, p = 0.025)
Budd Haeberlein et al	2022	20 countries (not specified)	Randomised, double-blind, placebo-controlled trials	1638 participants in EMERGE and 1647 in ENGAGE	1:1:1 low-dose, high-dose aducanumab, or placebo every 4 weeks Duration: 76 weeks (20 doses total)	Clinical Dementia Rating–Sum of Boxes (CDR-SB)	EMERGE: high-dose of aducanumab was effective in reducing CDR-SB scores (mean difference -0.39; 95% CI − 0.69 to − 0.09; 22% decrease, for high-dose vs placebo) Statistically significant improvement in men but not in women: CDR-SB scores adjusted mean difference -0.57 in men versus -0.21 in women. Greater differences on secondary outcomes between intervention and placebo in men ENGAGE: none of the differences between aducanumab and placebo were statistically significant. No significant difference between men and women. CDR-SB scores adjusted mean difference -0.21 in men versus 0.21 in women
Van Dyck et al	2023	Multicentre (not specified)	Phase III randomised, double-blind, placebo-controlled trial	1795 participants (898 lecanemab, 897 placebo)	1:1 intravenous lecanemab every 2 weeks or placebo Duration: 18 months	Clinical Dementia Rating–Sum of Boxes (CDR-SB)	Lecanemab was more effective compared to placebo More effective in men than in women CDR-SB scores adjusted mean difference -0.73 (43% slowing of decline) in men versus -0.2 (12% slowing of decline) in women. Greater differences on secondary outcomes between intervention and placebo in men
Cohen et al	2023	Multicentre (not specified)	Phase III randomised, double-blind, placebo-controlled trial	1795 participants (898 lecanemab, 897 placebo)	1:1 intravenous lecanemab every 2 weeks or placebo Duration: 18 months	HRQoL (measured with EQ-5D-5L and QOL-AD)	Lecanemab was effective in maintaining HRQoL (EQ-5D-5L adjusted mean difference 2.02, p = 0.004. QoL-AD adjusted mean treatment difference 0.66, p = 0.002) No statistically significant difference in EQ-5D-5L scores between sexes, but significantly more effective in men than women according to QOL-AD scores (31% less decline for men and 14% for women)

*CI* Confidence Interval, *CST* Cognitive Stimulation Therapy, *HRQoL* Health-Related Quality of Life, *CDR-SB* Clinical Dementia Rating–Sum of Boxes, *QOL-AD* Quality of Life in Alzheimer’s Disease

Preferred Reporting Items for Systematic Reviews and Meta-Analyses (PRISMA) flow diagram illustrating the study selection process for the scoping review. The initial search was conducted in November 2023 and updated in January 2024.

### Characteristics of included studies

Table 1 presents the characteristics of studies focusing on sex and gender differences in access to treatment, monitoring and management of dementia. The outcomes of these studies include sex and gender differences in anti-dementia medication prescribing, psychotropic and antipsychotic drug prescribing initiation and duration, quality of care, and use of inappropriate medications. Table 2 shows studies examining the effectiveness of treatments in reducing cognitive decline. These studies involve randomised controlled trials (RCTs) and explored outcomes such as cognitive decline, clinical dementia rating scores and health-related quality of life (HRQoL).

### Access to treatment and quality of care

Two studies examined gender differences in prescription and receipt of anti-dementia medications (AChEIs and memantine): one in the US [40] and one in the UK [27], and reported different results. Cooper et al. [27] found that, in the UK, women with dementia were *less* likely than men to be prescribed anti-dementia drugs. In contrast, Lu et al. [40] found that female Medicare beneficiaries in the US with AD and related dementias were 1.7 times *more* likely than males to receive anti-dementia medications. This difference was greater in people with AD-related dementias, but not significant when comparing only those with AD (See Table 1).

Regarding differences in primary care, women in the UK received significantly lower rates of GP consultations, annual blood pressure monitoring, and annual weight monitoring compared to men [29]. Outside the UK, sex and gender differences in changes in quality of dementia care were examined after implementation of the Quebec Alzheimer’s Plan (QAP), a subnational primary care policy intervention [28]. Quality of care was measured by a score based on several domains that followed national recommendations in Canada for dementia treatment and management [28]. While overall improvements in dementia care quality were observed after QAP implementation, the improvements were greater for men than for women.

Several studies have examined differences in polypharmacy between men and women with dementia, with mixed results. Two studies [29, 41] used the same UK dataset but focused on different cohorts. Cooper et al. [29] found, that between 2002 and 2013, women with dementia living outside long-term care facilities were less likely than men to be prescribed any type of psychotropic or anxiolytic medication. However, once prescribed, women were more likely to use these medications for longer compared to men. Analysing prescriptions between 2014 and 2016 and including people living both in the community and long-term care settings, women were less likely than men to be taking antipsychotics, but there was no significant difference in the use of anxiolytics [41]. In primary care, women with dementia without a diagnosis of a psychotic disorder were more likely to receive repeated antipsychotic prescriptions compared to men, although this difference was not statistically significant [42]. As noted earlier, antipsychotic medications are generally not recommended for people with dementia because of their side effects. In contrast, in inpatient settings, men with dementia were significantly more likely to be prescribed antipsychotics than women [43].

Two further studies examined the use of inappropriate medication in people with dementia. Tjia et al. [44] investigated nursing home residents with advanced dementia in the US and Montastruc et al. [45] focused on people living at home cared for by unpaid carers in France. Both studies showed that more than 40% of the samples of people with dementia were prescribed at least one inappropriate medication. However, regarding sex and gender differences, the studies showed different results. Tjia et al. [44] found that men in nursing homes were significantly more likely to use inappropriate medication, as classified by Holmes et al. [46], whereas Montastruc et al. [45] found that women living at home were significantly more likely to use potentially inappropriate medication identified by the LaRoche list [47].

### Treatment effectiveness

Some studies have explored the role of physical exercise as a treatment for improving cognition or slowing cognitive decline, potentially through mechanisms such as increased blood flow to the brain, reduced inflammation, and enhanced neuroplasticity. Lamb et al. [48] assessed the impact of moderate to high-intensity exercise training on cognitive decline in people with dementia over 12 months, using the 11-item cognitive subscale of the AD Assessment Scale (ADAS-Cog11). People were randomised to either (a) a tailored and supervised exercise programme twice a week for 4 months, followed by a home-based, unsupervised programme of 150 min of exercise per week, or (b) usual care according to clinical guidelines. The overall results showed that moderate to high-intensity exercise was not effective in slowing cognitive decline in people with dementia, and there was no difference between women and men when comparing treatment versus usual care. In contrast, Baker et al. [49] found that, after 6 months, a high-intensity aerobic intervention improved cognition (as measured by tests of executive function and short-term memory) in men and women with mild cognitive impairment, compared to a stretching control group. The disaggregated data showed a greater impact on executive function, including selective attention, search efficiency, processing speed, and verbal fluency, in women compared to men.

Cognitive stimulation therapy (CST) is a non-pharmacological intervention designed to enhance cognitive and social functioning by stimulating cognitive abilities [50]. A 7-week CST intervention resulted in improved cognition and quality of life in people with dementia, regardless of AChEI medication use [50]. The study showed that the cognitive benefits were significantly associated with female gender and older age, with women experiencing greater improvements than men in communication, social interactions, and quality of life.

We found only one study examining the effectiveness of anti-dementia drugs that provided disaggregated data by sex [51]. This study assessed the impact of nilvadipine on the rate of cognitive decline in people with mild to moderate AD. The primary outcome was the change in ADAS-Cog12 scores after 78 weeks of nilvadipine treatment compared to placebo. The overall results showed no differences in ADAS-Cog12 between the groups. However, when analysing the results by sex, men showed less decline than women on nilvadipine compared to placebo. The study evaluated other secondary outcomes but did not provide disaggregated results by sex.

Sex and gender differences have been reported in the supplementary material of two clinical trials of DMTs [52, 53]. The Clarity-AD study examined the effectiveness of lecanemab versus placebo in people with early AD [52]. The primary outcome was the change in the Clinical Dementia Rating–Sum of Boxes (CDR-SB) from baseline to 18 months. Other outcomes included changes in amyloid burden, ADAS-Cog14, AD Composite Score (ADCOMS) and AD Cooperative Study–Activities of Daily Living Scale for Mild Cognitive Impairment (ADCS-MCI-ADL). The results showed that lecanemab was moderately effective in reducing decline on measures of cognition and function, and in reducing amyloid burden in early AD; however, it was associated with increased adverse events such as infusion-related reactions, atrial fibrillation, and an increased risk of fainting (syncope). Analysis of the supplementary material and disaggregated data on measures of cognition and function showed that lecanemab appeared more effective in men than in women compared to placebo at 18 months [52]. Nevertheless, there was some overlap on the confidence intervals between men and women across all measures compared (CDR-SB, ADAS-Cog14, ADCOMS, ADCS-MCI-ADL) highlighting the need for further research to reach more definitive conclusions.

Another study using data from the Clarity-AD trial showed that people treated with lecanemab experienced reduced decline in quality of life compared to those receiving placebo, as measured by the European Quality of Life–5 Dimensions (EQ-5D-5L) and Quality of Life in AD (QOL-AD) [54]. The study showed that lecanemab reduced the decline in EQ-5D-5L scores for both males and females, with no significant difference between the sexes (48% less decline for males and 50% less decline for women in the lecanemab arm). The QOL-AD scores showed that lecanemab was more effective in preserving quality of life related to dementia in men than in women (31% less decline for men and 14% less decline for women).

Aducanumab was evaluated in terms of efficacy and safety in early AD in the EMERGE and ENGAGE studies [53]. Participants were randomly assigned to receive aducanumab at low dose, high dose, or placebo. The primary outcome was change in the CDR-SB from baseline to week 78. Secondary outcomes were the Mini-Mental State Examination (MMSE), ADAS-Cog13 and ADCS-MCI-ADL scores. The authors reported that high-dose of aducanumab effectively reduced CDR-SB scores in the EMERGE study, but not in the ENGAGE [53]. Both studies provided disaggregated data by sex for comparing the high-dose group versus placebo. The EMERGE study showed that, in general, aducanumab was more effective in males than females, although some differences were not statistically significant. In the ENGAGE study, none of the differences between aducanumab and placebo were statistically significant, and there was no difference in cognitive decline between men and women.

## Discussion

Our scoping review aimed to synthesise the evidence on sex and gender differences in dementia, focusing on differences in access, quality of care, and effectiveness of treatments. Some studies reporting inequalities in access and monitoring of the disease showed disadvantages for women with dementia in the UK: compared to men, they are more likely to be prescribed psychotropic medication for longer, receive fewer healthcare consultations and, ultimately, less health monitoring, although this was not consistently the case across all studies [27, 29]. These differences in prescribing attitudes might significantly impact health outcomes and increase the risk of adverse events. In addition, reduced healthcare contacts limit opportunities to review and mitigate inappropriate medication use.

These differences can be partially explained by women often being diagnosed at later stages of dementia and less likely to receive an accurate diagnosis compared to men [29, 55]. Also, women are usually better at masking dementia symptoms and perform better on cognitive tests [56]. In addition, women are more likely to live alone in later life, potentially leading to delayed recognition of dementia symptoms [57–59]. Gender differences in access to diagnosis and treatment can be influenced by social roles and cultural dynamics, which may also explain some of the observed differences [60]. Finally, some differences in access to treatment and care might arise because men are more often accompanied to healthcare settings by a carer, typically their spouse [61]. In contrast, women, who are more likely to live alone in later life, may encounter greater difficulties accessing treatment and care [29].

In some settings, men were more likely to be initiated on antipsychotic medications, whereas women stayed on them for longer periods [29], but again this pattern was not seen across all studies. This difference might be due to men presenting more aggressive behaviours, leading to medication prescription [62, 63]. On the other hand, aggression is often perceived as less socially acceptable for women than for men [62], potentially leading to a lower threshold for prescribing antipsychotics to women, and for longer duration [29]. Prolonged prescription of certain treatments, such as antipsychotics, is considered a sign of poor quality of care, as most of these medications are not licensed or recommended for extended use in older populations [64, 65].

Studies of the impact of exercise on cognitive decline yielded mixed results [48, 49], and limited disaggregated data by sex indicated a more pronounced impact in women than in men [49]. In addition, a CST intervention study also showed greater cognitive improvements for women [50].

The majority of clinical trials of DMTs for AD did not analyse potential sex and gender differences in key outcomes, despite some studies indicating that DMTs may have different effects on males and females. Recently, dementia experts have emphasised the need to explore sex and gender differences in AD clinical trials, ensuring that future trials include sex-disaggregated data and consider potential sex-specific treatment effects [66].The initial findings from sub-analyses of the clinical trials suggest that women may be less responsive to DMTs than men [12]. The reasons behind this potential disparity are not well understood, and so these results need to be considered with caution. Potential factors contributing to these differences include variations in amyloid levels in the brain, specific comorbidities, and additional biological factors such as hormones, pregnancy, and menopause [67–69]. To provide more conclusive evidence on sex and gender differences in the effectiveness of DMTs for dementia, clinical trials need to be adequately powered to analyse and report sex-disaggregated outcomes [70]. This will enable further examination of potential sex differences in pharmacokinetics and pharmacodynamics of new and existing agents and ensure that the use of these treatments does not increase gender disparities [71].

There are several challenges associated with the costs and administration burden of DMTs, potentially increasing health inequalities. Due to their current eligibility criteria, DMTs may initially only be accessible to a few relatively fit individuals in specialist centres. Also, given the high burden of administering the drugs and frequent appointments required, these new treatments might disadvantage people who live alone, live far from specialist centres, do not have support from a carer, and, within the UK context, do not speak English as their first language. Therefore, a gender difference could be expected: men are more likely to be supported by their carer or spouse, whereas women are more likely to live alone and enter care homes [72], due to their longer life expectancy. To reduce barriers to access and address these disparities, services providing DMTs will need to address these potential inequalities, and the associated increased burden for some individuals and their carers [73].

We found some evidence of sex and gender differences for some treatments, activities and programmes [48–50, 52–54], but many studies are not designed to investigate sex- and gender-related differences in effectiveness. Usually, subgroup analyses by sex and gender were not the primary objectives and were only reported, if at all, in the supplementary material of the published papers. There is a need to collect more detailed epidemiological, clinical, and other research data to better understand how health inequalities impact treatment effectiveness by sex and gender.

## Strengths and limitations

A comprehensive search was conducted following PRISMA guidelines, ensuring a systematic and transparent approach to literature identification and selection. The search strategy across multiple databases and grey literature sources enhanced the breadth and robustness of the findings. Additionally, manually retrieving references from relevant studies further broadened the review scope. Study selection and data extraction processes involved multiple reviewers, reducing the risk of bias and ensuring accuracy and reliability.

Despite these strengths, the review has limitations. The initial restriction to UK-based studies, although later expanded, may have affected the generalisability of the findings, particularly in capturing global sex and gender differences. Identifying studies with sex-disaggregated data proved challenging, as the terms "sex" and "gender" were often absent from titles and abstracts. As a result, supplementary materials and appendices frequently had to be consulted. Another limitation is the variability in study designs, methodologies, and outcome measures, which complicates direct comparisons and synthesis of findings. For example, differences in dementia care quality, medication prescribing, and treatment effectiveness were assessed using diverse metrics, making it difficult to draw definitive conclusions about sex and gender disparities. Lastly, while this review highlights important sex and gender differences in dementia management and treatment, it does not systematically explore the underlying biological, social, and healthcare system factors contributing to these disparities. Future research should integrate qualitative and quantitative data to provide a more comprehensive understanding of the mechanisms driving these differences.

## Conclusions

Addressing sex and gender differences in dementia care is crucial for ensuring equitable access to diagnosis, treatments, quality of care, and effectiveness of treatments for both men and women. These differences should be considered when planning and implementing healthcare, social care, and associated strategies in dementia. Both men and women should have equal access to timely and accurate dementia diagnosis, and current disparities should be addressed. Treatment approaches should be personalised and take into account that men and women may respond differently to therapies and medications. Care quality should be improved by addressing sex and gender differences, such as in health monitoring, and healthcare professionals should be trained accordingly. Clinical trials should include well-powered samples and report sex and gender differences to accurately assess treatment effectiveness for both men and women. Funding bodies should prioritise research funding for studies investigating sex and gender differences in dementia to better inform future policy. Addressing these implications will help provide more equitable and effective care for both men and women living with dementia.

## Supplementary Information

Supplementary Material 1.

## Data Availability

No datasets were generated or analysed during the current study.

## Abbreviations

AChEIs: Acetylcholinesterase Inhibitors
AD: Alzheimer’s Disease
ADAS-Cog: Alzheimer’s Disease Assessment Scale – cognitive subscale
ADCOMS: Alzheimer’s Disease Composite Score
ADCS-ADL-MCI: Alzheimer’s Disease Cooperative Study—Activities of Daily Living Scale for use in Mild Cognitive Impairment
CDR-SB: Clinical Dementia Rating scale – Sum of Boxes
CI: Confidence Interval
CPRD: Clinical Practice Research Datalink
CST: Cognitive Stimulation Therapy
DMT: Disease Modifying Treatment
EMA: European Medicine Agency
EQ-5D-5L: European Quality of Life–5 Dimensions
FDA: Food and Drug Administration
HRQoL: Health Related Quality of Life
IRR: Incidence Rate Ratio
MHRA: Medicines and Healthcare products Regulatory Agency
MMSE: Mini-Mental State Examination
OR: Odds Ratio
PIM: Potentially Inappropriate Medications
PRISMA: Preferred Reporting Items for Systematic Review and Meta-Analysis
PRR: Prevalence Rate Ratio
QAP: Quebec Alzheimer’s Plan
QOL-AD: Quality of Life in Alzheimer’s Disease
RCT: Randomized Controlled Trial
THIN: The Health Improvement Network
