# Supplementary material for: Sex and gender differences in access, quality of care, and effectiveness of treatment in dementia: a scoping review of studies up to 2024
Source: Arch Public Health. 2025 May 29;83:139. doi: 10.1186/s13690-025-01626-z (PMC12121192; doi:10.1186/s13690-025-01626-z)
Supplement: Supplementary file 1 — Supplementary Material 1. [file 13690_2025_1626_MOESM1_ESM.docx]

**Supplementary Table 1: Search criteria by database for studies published up to January 2024 (no start date applied)**

| **Database** | **Search** | **Search terms** |
| --- | --- | --- |
| Medline | 1 | exp dementia/ or exp Alzheimer Disease/ |
|  | 2 | disease modifying treatment*.mp. |
|  | 3 | cognitive stimulation therapy.mp. |
|  | 4 | clinical trials.mp. |
|  | 5 | ((treatment or care) and (effectiveness or access or quality)).mp. |
|  | 6 | medication.mp. |
|  | 7 | physical activity.mp. |
|  | 8 | ((sex or gender) and difference).mp. |
|  | 9 | 2 or 3 or 4 or 5 or 6 or 7 |
|  | 10 | 1 and 8 and 9 |
| EMBASE | 1 | exp dementia/ or exp Alzheimer Disease/ |
|  | 2 | disease modifying treatment*.mp. |
|  | 3 | cognitive stimulation therapy.mp |
|  | 4 | clinical trials.mp. |
|  | 5 | ((treatment or care) and (effectiveness or access or quality)).mp. |
|  | 6 | medication.mp. |
|  | 7 | physical activity.mp. |
|  | 8 | ((sex or gender) and difference).mp. |
|  | 9 | 2 or 3 or 4 or 5 or 6 or 7 |
|  | 10 | 1 and 8 and 9 |
| PubMed | 1 | (lecanemab[Title/Abstract]) OR (aducanumab[Title/Abstract]) |
